# Abstracts and Selections

**Published:** 1909-11

**Authors:** 


### Abstracts and Selections.

---

Dr. G. Frank Lydston, of Chicago, one of the most distinguished members of the American medical profession, came to Columbus Monday, at the invitation of the North Side Medical Research Society, and at a dinner given at the Northern Hotel in the evening, he explained in his inimitable way the basis of his campaign against what he describes as an inside clique that is dominating the affairs of the American Medical Society. This lecture was heard by 150 Columbus and Central Ohio physicians and others interested, and a rousing vote of thanks was tendered to Dr. Lydston after he had concluded his address.

### WORKING FOR A REFORM.

Contrary to the general impression as to Dr. Lydston's intentions, he is not attacking the American Medical Association in an effort to disrupt it or work damage to its purposes, but to correct what he believes to an evil, and to place the control of the organization in the hands of the members, where he says it belongs, instead of allowing the society to continue to be dominated by the "inside clique," of which the Secretary, Dr. Simmons, is the head. Dr.

Lydston has given largely of his personal means and time to his campaign, and has succeeded in awakening the profession almost throughout the entire United States to at least a keen interest in what he is attempting to bring about.

BASIS OF COMPLAINT.

Dr. Lydston said in part: "The reorganization of the American Medical Association in 1902 was very attractive to those who did not delve beneath the surface where the alleged object of unifying and fostering the welfare of the medical profession was corrupted by the fine Italian hand of the medical politician and monopolist.

"The machine thus formed," continued Dr. Lydston, "has developed into one of the most selfish and monopolistic political organizations in the country. The American Medical Association at present is a paradoxical combination of trust monopoly and trades unionism."

SEES DANGER AHEAD.

The speaker also charged that the old ideas of the profession had been laid aside, and that the association has developed into a huge business, the foundation stones of which are the acquirement of wealth and the development of unlimited political power.—From *Columbus* (Ohio) *Evening Dispatch*, October 5, 1909.

